# Supplementary material for: A Likelihood Approach for Real-Time Calibration of Stochastic Compartmental Epidemic Models
Source: PLoS Comput Biol. 2017 Jan 17;13(1):e1005257. doi: 10.1371/journal.pcbi.1005257 (PMC5240920; doi:10.1371/journal.pcbi.1005257)
Supplement: S1 File — (TAR.GZ) [file pcbi.1005257.s014.tar.gz › HSPH_Online-SI-Revision/EnKF07/n1-severe/n1-severe_EnKF_table0.pdf]

1	R0	{1.51081, 13.7078}	{2.8007, 5.17259}	{{2.4441, 1.78274}, {3., 9.90684}}	{85.3773, 62.5393}	inf	54.	52.7119	{6.22658, 45.329}	104.331	0.481717	newly	34.	60.85	{5., 64.}	150.912	0.409655	Reff	1.05303	0.500378	{0.174144, 0.819232}	60.8564	ar	656.	1739.76	{1612., 1772.}	165.207
2	R0	{1.73182, 11.3873}	{2.61583, 6.6142}	{{1.7913, 3.13135}, {3., 14.2873}}	{52.6695, 52.3115}	inf	80.	66.854	{7.30973, 103.15}	112.551	0.647765	newly	53.	66.11	{5., 190.}	136.245	0.593862	Reff	1.15166	0.491398	{0.0660489, 0.913361}	63.903	ar	653.	1813.	{1610., 2292.}	177.642
3	R0	{1.50449, 10.3474}	{2.29601, 7.33646}	{{0.934204, 2.29923}, {3., 10.2925}}	{74.9672, 32.5798}	inf	60.	68.3802	{4.05879, 108.776}	128.155	0.60517	newly	43.	63.41	{2., 109.}	158.442	0.65935	Reff	1.08474	0.471787	{0.0561617, 0.848654}	63.1625	ar	651.	1806.87	{1619., 2264.}	177.553
4	R0	{1.63493, 9.1955}	{2.23752, 5.12549}	{{0.630172, 1.77703}, {3., 10.5238}}	{71.4222, 50.1441}	inf	87.	59.4099	{9.03436, 74.4605}	87.012	0.562548	newly	59.	70.67	{7., 71.}	114.831	0.472661	Reff	1.05126	0.406985	{0.0897249, 0.657492}	64.2461	ar	663.	1841.35	{1679., 2062.}	177.73
5	R0	{1.53822, 8.05553}	{2.63249, 7.34508}	{{2.24622, 0.791956}, {3., 16.8419}}	{77.0519, 62.2874}	inf	55.	46.2295	{4.0792, 91.4114}	88.1963	0.509205	newly	40.	82.09	{4., 62.}	199.975	0.514616	Reff	1.01061	0.531504	{0.148295, 0.8197}	62.8874	ar	621.	1791.21	{1630., 1885.}	188.44
6	R0	{1.69374, 17.0793}	{2.8125, 7.85233}	{{2.40568, 1.2042}, {3., 17.2457}}	{66.0529, 54.8757}	inf	97.	43.4232	{9.42103, 51.8077}	95.2293	0.762016	newly	40.	51.79	{4., 33.}	149.025	0.574618	Reff	1.25676	0.513298	{0.119683, 0.590239}	68.9191	ar	662.	1709.03	{1552., 2192.}	158.162
7	R0	{1.42179, 11.8031}	{2.77687, 6.94781}	{{2.40263, 1.03788}, {3., 14.3384}}	{95.3078, 52.597}	inf	57.	33.6707	{8.05601, 34.1322}	84.5634	0.55669	newly	30.	58.72	{6., 45.}	185.467	0.481891	Reff	0.963974	0.504681	{0.122683, 0.953216}	63.1093	ar	517.	1713.44	{1574., 2116.}	231.42
8	R0	{1.46683, 6.95875}	{2.3252, 5.55051}	{{0.535572, 1.06889}, {3., 11.4774}}	{89.0212, 54.5575}	inf	92.	66.985	{9.73715, 101.639}	89.1788	0.534421	newly	97.	77.09	{7., 81.}	101.495	0.611097	Reff	0.815557	0.417078	{0.0832227, 0.642051}	53.2451	ar	653.	1817.61	{1662., 1938.}	178.348
9	R0	{1.34525, 11.505}	{2.77588, 7.32558}	{{2.40865, 1.25999}, {3., 14.4309}}	{106.347, 52.4644}	inf	47.	35.5352	{13.4889, 54.8442}	87.0525	0.475146	newly	31.	58.34	{6., 56.}	184.065	0.500159	Reff	1.01701	0.522672	{0.137223, 0.70441}	62.9422	ar	532.	1710.17	{1560., 2070.}	221.461
10	R0	{1.77958, 14.372}	{2.80432, 7.76348}	{{2.39842, 3.64369}, {3., 12.1406}}	{57.5835, 48.0656}	inf	110.	49.1428	{9.00681, 48.6022}	93.4775	0.75851	newly	47.	59.92	{4., 86.}	147.787	0.634197	Reff	0.948516	0.59432	{0.151449, 1.42565}	66.9988	ar	671.	1755.86	{1564., 2350.}	161.678
11	R0	{1.60334, 19.9548}	{2.74397, 3.08209}	{{2.47466, 1.00043}, {3., 11.6213}}	{74.1846, 84.5546}	inf	94.	35.4807	{5.51092, 46.4239}	82.2713	0.724192	newly	31.	46.46	{6., 68.}	104.452	0.347356	Reff	1.08867	0.44163	{0.205429, 0.79295}	64.121	ar	617.	1647.25	{1533., 1765.}	166.977
12	R0	{1.68623, 7.58585}	{2.39757, 6.00014}	{{1.65754, 3.01572}, {3., 9.47932}}	{44.5948, 42.4704}	inf	74.	72.508	{7.5842, 230.439}	113.001	0.578808	newly	59.	88.66	{4., 177.}	160.102	0.595755	Reff	0.881898	0.524451	{0.100605, 1.64993}	63.7116	ar	640.	1881.88	{1655., 2490.}	194.044
13	R0	{1.82689, 18.0022}	{2.802, 7.43166}	{{2.40251, 3.63932}, {3., 15.4752}}	{53.3753, 58.8467}	inf	136.	37.538	{13.0247, 33.3678}	86.8015	0.837112	newly	49.	45.78	{6., 38.}	118.408	0.603906	Reff	1.10344	0.512732	{0.133712, 0.741898}	66.2997	ar	673.	1724.47	{1569., 2199.}	156.236
14	R0	{1.75911, 9.16883}	{2.351, 5.86347}	{{1.62069, 2.92006}, {3., 8.62187}}	{42.896, 42.915}	inf	93.	74.9661	{3.7552, 171.284}	108.688	0.706101	newly	75.	91.92	{4., 173.}	143.84	0.711557	Reff	1.16629	0.527097	{0.0518819, 1.42357}	67.8431	ar	661.	1895.1	{1637., 2743.}	186.702
15	R0	{1.46168, 11.3859}	{2.62355, 6.35571}	{{1.97092, 3.19168}, {3., 8.75384}}	{79.4884, 44.8478}	inf	70.	50.4159	{7.25581, 28.3062}	102.793	0.611751	newly	36.	42.5	{5., 46.}	112.778	0.472734	Reff	0.809771	0.464747	{0.11237, 0.857575}	60.5631	ar	556.	1674.57	{1543., 1931.}	201.182
16	R0	{1.53577, 11.7581}	{2.40994, 6.34622}	{{1.62543, 3.78873}, {3., 9.79621}}	{58.3307, 49.4433}	inf	94.	86.3206	{6.13397, 248.631}	114.29	0.656894	newly	55.	96.37	{5., 237.}	177.182	0.579882	Reff	1.05507	0.501291	{0.0612153, 0.923708}	65.6201	ar	625.	1865.31	{1633., 2373.}	198.45
17	R0	{1.70698, 9.72924}	{2.30661, 6.66168}	{{1.6087, 3.53947}, {3., 12.5684}}	{44.4009, 43.3735}	inf	101.	73.6282	{7.66884, 135.48}	94.7433	0.610798	newly	63.	77.25	{7., 115.}	124.079	0.540931	Reff	0.913234	0.507838	{0.101167, 1.12904}	61.2801	ar	675.	1897.24	{1651., 2440.}	181.073
18	R0	{1.5973, 14.931}	{2.47592, 6.7018}	{{1.64332, 3.42252}, {3., 9.61055}}	{60.7889, 55.4212}	inf	53.	50.5859	{9.53649, 67.5368}	84.4894	0.40425	newly	23.	55.1	{9., 59.}	181.043	0.326511	Reff	1.28583	0.45825	{0.0397093, 0.637045}	68.3987	ar	632.	1811.34	{1654., 2132.}	186.604
19	R0	{1.87468, 17.004}	{2.5431, 9.18457}	{{1.25816, 3.33217}, {3., 18.401}}	{51.1237, 48.8297}	inf	112.	37.654	{13.6147, 50.259}	75.2662	0.62632	newly	45.	28.98	{6., 43.}	74.1778	0.483159	Reff	0.987956	0.393477	{0.166404, 0.542821}	64.8616	ar	692.	1631.2	{1546., 1637.}	135.723
20	R0	{1.84715, 12.531}	{2.40148, 3.64588}	{{1.13856, 0.61785}, {3., 4.40845}}	{52.3267, 71.8672}	inf	90.	52.3682	{4.22985, 94.4963}	94.4211	0.71613	newly	55.	72.56	{8., 88.}	132.982	0.510675	Reff	0.936505	0.414562	{0.175345, 0.649815}	58.0148	ar	661.	1661.73	{1525., 1838.}	151.396
21	R0	{1.75938, 13.5959}	{2.42362, 6.38981}	{{1.61545, 2.56659}, {3., 11.923}}	{45.0169, 53.0019}	inf	92.	84.836	{10.1009, 210.611}	104.082	0.558403	newly	50.	93.51	{8., 172.}	164.78	0.456401	Reff	1.1911	0.526899	{0.0622124, 1.04667}	63.934	ar	620.	1900.18	{1652., 2649.}	206.481
22	R0	{1.63456, 10.7469}	{2.64267, 4.16379}	{{1.94812, 2.02056}, {3., 5.53905}}	{61.675, 62.3812}	inf	85.	34.8781	{7.16733, 62.9886}	78.9406	0.679962	newly	61.	59.7	{5., 72.}	115.508	0.61355	Reff	0.81728	0.476881	{0.0939038, 0.624813}	60.9543	ar	654.	1682.84	{1547., 2036.}	157.315
23	R0	{1.62702, 19.1392}	{2.33211, 6.24041}	{{0.924614, 1.40331}, {3., 12.9864}}	{67.695, 67.3946}	inf	97.	63.1742	{6.41313, 53.57}	104.109	0.717775	newly	37.	66.58	{5., 87.}	169.784	0.503335	Reff	1.02014	0.434192	{0.118355, 0.755806}	62.9524	ar	600.	1791.8	{1622., 1990.}	198.633
24	R0	{1.70466, 12.1202}	{2.49254, 8.44297}	{{1.25205, 3.11716}, {3., 18.3164}}	{59.6025, 53.5446}	inf	70.	46.8841	{12.4535, 49.1659}	79.3074	0.466036	newly	32.	38.5	{7., 56.}	94.5625	0.358266	Reff	0.946086	0.455714	{0.103363, 0.679687}	61.9677	ar	613.	1720.38	{1587., 1997.}	180.649
25	R0	{1.61735, 11.3169}	{2.69548, 4.56107}	{{2.138, 2.52967}, {3., 5.22091}}	{67.3909, 64.9752}	inf	103.	52.047	{9.15658, 51.339}	90.8179	0.689702	newly	62.	68.52	{9., 137.}	111.419	0.491758	Reff	0.826466	0.563783	{0.092078, 1.10124}	56.2846	ar	681.	1691.89	{1536., 1984.}	148.442
26	R0	{1.56977, 12.3035}	{2.62348, 6.70931}	{{2.24596, 0.652276}, {3., 14.6137}}	{75.798, 55.5983}	inf	78.	36.4542	{3.23329, 55.8341}	82.3043	0.710813	newly	42.	58.53	{2., 49.}	151.976	0.597197	Reff	1.03605	0.497773	{0.0867401, 0.805593}	62.9057	ar	594.	1757.62	{1626., 1856.}	195.896
27	R0	{1.46317, 5.8298}	{2.70339, 7.37214}	{{1.83114, 3.40708}, {3., 17.0091}}	{84.7623, 66.1642}	inf	64.	53.3801	{9.13272, 94.9158}	89.2805	0.489164	newly	64.	61.77	{7., 148.}	115.672	0.615585	Reff	0.847175	0.580138	{0.102175, 1.18306}	57.8232	ar	586.	1788.07	{1626., 2029.}	205.131
28	R0	{1.70002, 14.9806}	{2.39541, 6.35845}	{{1.6299, 3.34696}, {3., 10.7172}}	{44.5379, 57.5554}	inf	110.	74.2528	{6.23672, 139.637}	101.965	0.712268	newly	42.	81.62	{4., 156.}	179.19	0.507538	Reff	1.16281	0.484805	{0.0419996, 1.19162}	69.701	ar	652.	1864.36	{1623., 2369.}	185.945
29	R0	{1.52729, 16.1014}	{2.51738, 5.97208}	{{1.18383, 3.25289}, {3., 8.18739}}	{75.4405, 62.9096}	inf	86.	67.5159	{4.25044, 92.3203}	99.9913	0.624541	newly	44.	78.53	{4., 212.}	162.659	0.503846	Reff	0.925538	0.519836	{0.180787, 0.703439}	57.9825	ar	592.	1749.79	{1545., 2509.}	195.573
30	R0	{1.72795, 13.5635}	{2.65485, 6.20912}	{{1.96244, 0.712215}, {3., 15.2751}}	{53.6416, 60.0388}	inf	92.	48.2113	{10.3588, 86.2648}	58.8257	0.488029	newly	43.	67.33	{9., 96.}	125.884	0.407542	Reff	0.865703	0.478844	{0.0508367, 0.929727}	63.8896	ar	668.	1730.65	{1589., 1870.}	159.079
31	R0	{1.51591, 18.1544}	{2.80061, 5.0812}	{{2.4433, 3.59567}, {3., 6.05773}}	{84.748, 72.0112}	inf	59.	55.1978	{3.98269, 49.1688}	118.719	0.612716	newly	16.	62.28	{4., 52.}	332.125	0.385203	Reff	1.1051	0.488674	{0.135881, 0.572475}	64.7037	ar	541.	1736.07	{1575., 1864.}	220.9
32	R0	{1.4282, 10.3534}	{2.57328, 5.82077}	{{1.81367, 1.89977}, {3., 15.0389}}	{85.59, 60.8}	inf	38.	43.668	{5.56981, 54.0976}	106.469	0.439694	newly	21.	57.95	{7., 53.}	230.714	0.358222	Reff	1.1397	0.501527	{0.125106, 1.0969}	60.6966	ar	521.	1756.72	{1610., 1978.}	237.182
33	R0	{1.56693, 11.3972}	{2.32385, 6.51008}	{{1.61421, 2.61591}, {3., 13.589}}	{55.9717, 49.1981}	inf	107.	77.0388	{7.47395, 113.14}	102.182	0.681846	newly	62.	76.98	{7., 103.}	130.484	0.584902	Reff	0.965229	0.498905	{0.041984, 1.01428}	61.8778	ar	695.	1850.83	{1640., 2409.}	166.306
34	R0	{1.50675, 19.8823}	{2.85126, 6.58748}	{{2.52019, 3.51339}, {3., 8.71057}}	{89.2324, 66.876}	inf	68.	81.7183	{22.7706, 101.003}	92.9541	0.335701	newly	22.	91.14	{16., 189.}	329.364	0.393999	Reff	0.90405	0.588003	{0.206649, 1.23472}	65.6651	ar	587.	1789.94	{1556., 2586.}	204.93
35	R0	{1.72889, 7.76068}	{2.37269, 6.84387}	{{1.60401, 3.45876}, {3., 12.5389}}	{46.0788, 45.6959}	inf	77.	72.1655	{5.50093, 160.965}	108.033	0.606973	newly	62.	79.07	{5., 226.}	141.21	0.66842	Reff	1.04771	0.518777	{0.06635, 1.24951}	63.5718	ar	650.	1894.44	{1650., 2419.}	191.452
36	R0	{1.62644, 4.12303}	{2.48077, 5.90025}	{{1.2528, 1.73941}, {3., 12.5733}}	{60.9059, 80.3531}	inf	43.	61.6242	{9.98973, 110.603}	81.0274	0.282314	newly	57.	54.69	{7., 95.}	67.0351	0.377748	Reff	0.769306	0.44485	{0.0784981, 0.852455}	64.1477	ar	652.	1740.68	{1588., 2112.}	166.975
37	R0	{1.70878, 15.8072}	{2.65464, 3.11407}	{{2.2971, 0.651281}, {3., 4.82976}}	{61.1835, 80.2997}	inf	84.	40.8696	{2.40254, 47.1975}	100.607	0.879417	newly	36.	93.84	{3., 139.}	257.889	0.584768	Reff	0.915906	0.57437	{0.203308, 1.07001}	64.9273	ar	674.	1682.2	{1526., 1886.}	149.585
38	R0	{1.58967, 6.80568}	{2.27228, 5.86005}	{{0.568458, 1.52096}, {3., 11.4541}}	{77.5228, 53.3631}	inf	73.	75.4295	{8.44986, 138.89}	104.204	0.492148	newly	63.	91.06	{7., 88.}	138.825	0.511212	Reff	0.86637	0.433964	{0.089442, 0.624328}	57.5294	ar	610.	1836.22	{1662., 1998.}	201.626
39	R0	{1.344, 8.19498}	{2.8099, 6.25636}	{{2.42707, 0.882067}, {3., 12.0535}}	{109.07, 50.2755}	inf	39.	46.8926	{5.30896, 56.8757}	117.185	0.477245	newly	36.	47.31	{4., 69.}	133.25	0.540684	Reff	0.928704	0.450245	{0.117304, 0.715502}	57.2838	ar	551.	1736.46	{1595., 2092.}	215.147
40	R0	{1.61509, 7.90825}	{2.64854, 5.48323}	{{2.15149, 1.06325}, {3., 10.6819}}	{65.6999, 47.8676}	inf	48.	66.7249	{10.0178, 113.531}	92.1397	0.295861	newly	41.	88.52	{9., 184.}	163.317	0.373487	Reff	1.04173	0.550948	{0.129652, 1.2665}	66.8329	ar	581.	1683.23	{670., 2230.}	196.408
41	R0	{1.60017, 5.90605}	{2.67346, 6.84854}	{{2.24675, 0.530184}, {3., 15.2235}}	{71.2642, 86.0357}	inf	76.	34.9002	{6.33464, 68.4797}	70.4985	0.588706	newly	76.	58.38	{6., 57.}	109.368	0.683013	Reff	0.832088	0.531211	{0.147556, 0.821386}	54.2163	ar	661.	1797.94	{1640., 2151.}	172.003
42	R0	{1.64436, 15.1337}	{2.68723, 6.21973}	{{2.2525, 0.652161}, {3., 15.0996}}	{67.5854, 62.8999}	inf	74.	50.89	{8.86438, 71.1708}	84.1082	0.54391	newly	39.	55.75	{8., 97.}	113.359	0.396483	Reff	1.11323	0.53305	{0.160959, 0.807972}	62.9976	ar	640.	1800.24	{1655., 1917.}	181.288
43	R0	{1.48917, 11.917}	{2.52205, 3.60746}	{{2.04163, 1.10894}, {3., 4.29412}}	{72.787, 77.9353}	inf	72.	35.9203	{2.35564, 39.1439}	102.015	0.862659	newly	36.	47.38	{3., 46.}	138.333	0.553359	Reff	0.927753	0.412097	{0.141698, 0.717903}	61.8222	ar	551.	1631.54	{1514., 1954.}	196.105
44	R0	{1.65577, 7.89317}	{2.29272, 5.33385}	{{0.585142, 1.66154}, {3., 11.0072}}	{71.3525, 50.1289}	inf	65.	56.0008	{9.96393, 82.9834}	88.6931	0.474286	newly	60.	63.79	{8., 69.}	102.583	0.474475	Reff	1.09446	0.425137	{0.088764, 0.642793}	66.4281	ar	576.	1811.95	{1670., 1964.}	214.575
45	R0	{1.6909, 12.3275}	{2.34955, 5.49588}	{{0.915105, 2.06334}, {3., 7.26088}}	{64.0045, 55.9488}	inf	82.	51.838	{7.81859, 76.1621}	80.1577	0.547698	newly	47.	75.46	{7., 133.}	148.936	0.482743	Reff	0.968886	0.508551	{0.0839565, 1.04478}	58.7733	ar	675.	1782.44	{1622., 2210.}	164.065
46	R0	{1.62351, 16.0698}	{2.78576, 6.89534}	{{2.41984, 1.42977}, {3., 13.908}}	{71.5886, 58.5343}	inf	58.	29.6592	{16.8166, 29.0134}	78.8197	0.53767	newly	28.	51.06	{7., 27.}	171.143	0.412629	Reff	1.25173	0.509728	{0.122957, 0.646435}	67.3002	ar	591.	1710.54	{1579., 2032.}	189.431
47	R0	{1.52372, 4.38599}	{2.45292, 5.71051}	{{1.12382, 0.756586}, {3., 9.20765}}	{69.4081, 58.3151}	inf	71.	65.7613	{17.5637, 127.07}	79.9826	0.397288	newly	95.	84.21	{12., 141.}	95.9263	0.547244	Reff	0.888329	0.578508	{0.157763, 1.5321}	60.9439	ar	618.	1816.88	{1623., 2406.}	195.184
48	R0	{1.44306, 11.788}	{2.80999, 6.81026}	{{2.42458, 0.710448}, {3., 11.0171}}	{94.7242, 45.6409}	inf	70.	43.2281	{15.3433, 46.1101}	83.8375	0.523067	newly	36.	49.39	{10., 59.}	123.472	0.402844	Reff	0.883153	0.456927	{0.18083, 0.682691}	53.3928	ar	534.	1709.88	{1591., 2037.}	220.202
49	R0	{1.63624, 11.3893}	{2.26784, 5.27036}	{{0.589227, 1.91967}, {3., 10.7574}}	{72.4353, 54.7829}	inf	91.	63.0896	{10.3058, 121.189}	90.9869	0.59294	newly	55.	69.64	{7., 72.}	112.836	0.449775	Reff	0.927748	0.409403	{0.0802264, 0.620117}	62.992	ar	643.	1824.57	{1666., 1984.}	183.759
50	R0	{1.46113, 11.1322}	{2.61208, 5.56042}	{{1.80471, 1.91104}, {3., 12.3286}}	{80.7086, 60.5453}	inf	63.	44.4964	{4.95419, 50.9589}	96.0335	0.627508	newly	52.	41.9	{3., 69.}	88.3462	0.554416	Reff	1.09731	0.484428	{0.0748495, 0.920924}	61.5449	ar	501.	1758.69	{1623., 2047.}	251.036
